# Even minor concomitant meniscus injuries are associated with posttraumatic osteoarthritis 15 years after anterior cruciate ligament reconstruction

**DOI:** 10.1002/jeo2.70440

**Published:** 2025-09-27

**Authors:** Padmini Naga Karamchedu, Anika N. Breker, Meggin Q. Costa, Gary J. Badger, Paul D. Fadale, Michael J. Hulstyn, Robert M. Shalvoy, Holly C. Gil, Tannin A. Schmidt, Braden C. Fleming

**Affiliations:** ^1^ University of Connecticut Health Center Farmington Connecticut USA; ^2^ Department of Orthopaedics, Rhode Island Hospital Brown University Providence Rhode Island USA; ^3^ Larner College of Medicine University of Vermont Burlington Vermont USA; ^4^ Department of Diagnostic Imaging, Rhode Island Hospital Brown University Providence Rhode Island USA; ^5^ Institute for Biology, Engineering and Medicine Brown University Providence Rhode Island USA

**Keywords:** ACL, meniscus, osteoarthritis, patient reported outcomes, risk factors

## Abstract

**Purpose:**

The aim was to evaluate the associations between baseline variables on the development of a symptomatic knee and imaging evidence of posttraumatic osteoarthritis (PTOA) 15 years after anterior cruciate ligament (ACL) surgery.

**Methods:**

Data from 42 subjects enroled in the Tension Trial (NCT00434837) were evaluated at 15‐year follow‐up. Patient sex, meniscus injury, preoperative patient‐reported outcomes (Knee Osteoarthritis Outcome Score [KOOS] function sports and recreation subscore [KOOS‐sport], Short Form [SF]‐36 mental health score), initial graft tension, and subsequent ACL surgery were evaluated to identify patients with a symptomatic knee using KOOS criteria (KOOS‐Quality of life ≤87.5 and with at least two other subscores meeting the following thresholds (i.e., KOOS‐Pain ≤86.1, KOOS‐Symptoms ≤85.7, KOOS‐Activities of daily living ≤86.8 and KOOS‐Sport ≤85.0) and imaging evidence of PTOA (Osteoarthritis Research Society International [OARSI] radiographic score and Whole‐Organ Magnetic Resonance Imaging Score [WORMS]) 15 years post‐surgery using stepwise regression.

**Results:**

The presence of a baseline meniscus tear was associated with a seven‐fold increase (*p* = 0.03) in odds for a symptomatic knee at 15 years. A higher preoperative KOOS‐sport was associated with a decreased occurrence of a symptomatic knee (*p* = 0.06). A higher KOOS‐sport and the presence of a meniscus tear at baseline were associated with a greater OARSI difference score (*p* = 0.03 and *p* = 0.05, respectively) 15 years after ACL reconstruction. The presence of the baseline meniscus tear was associated with a higher WORMS (*p* = 0.07) 15 years after ACL reconstruction. All other variables were not significant predictors, though loss to follow‐up was over 50%.

**Conclusion:**

The presence of a minor meniscus injury was associated with a higher occurrence of a symptomatic knee and imaging evidence of posttraumatic osteoarthritis 15 years after ACL reconstruction. Furthermore, a higher preoperative KOOS‐sport was associated with decreased occurrence of a symptomatic knee. Further studies are required to elucidate the associations of KOOS‐sport with imaging PTOA.

**Level of Evidence:**

Level IV.

**Abbreviations**
4‐ST/G: four‐stranded semitendinosus gracilis
ACL: anterior cruciate ligament
ADL: activities of daily living
AP: anteroposterior
BPTB: bone–patellar tendon–bone
KOOS: Knee Osteoarthritis Outcome Score
MR: magnetic resonance
MRI: magnetic resonance imaging
OA: osteoarthritis
OARSI: Osteoarthritis Research Society International
PTOA: posttraumatic osteoarthritis
QOL: quality of life
SD: standard deviation
SF‐36: Short Form 36
WORMS: Whole Organ MRI Score

## INTRODUCTION

Multiple factors are thought to contribute to the onset of posttraumatic osteoarthritis (PTOA) after anterior cruciate ligament (ACL) reconstruction surgery, including demographic, injury, and surgical variables [2, 20, 23, 29]. Factors that are predictive of a symptomatic knee and imaging evidence of knee PTOA remain a topic of debate [8]. Previous studies have utilised different patient reported outcomes as surrogates for a symptomatic knee, including the Knee Osteoarthritis Outcome Score (KOOS‐pain), KOOS‐quality of life (KOOS‐QOL) [12, 29] and the Marx activity score [22]. Englund et al. developed a composite model based on a combination of KOOS subscores to identify individuals symptomatic enough to seek medical care [13]. This model was selected for the current study as it captured the greatest number of symptomatic patients compared to other patient reported outcome models [29, 30].

There have been limited studies evaluating *long‐term* risk factors for both a symptomatic knee and imaging‐based PTOA after ACL reconstruction [19, 29, 30]. Patients with a low preoperative KOOS‐sport and low SF‐36 mental health outcome have been previously determined to have higher incidence of knee arthrosis 7 years after ACL surgery [22, 29]. At mid‐term follow‐up, patients who were symptomatic, as determined by KOOS‐pain, also had higher occurrence of PTOA on magnetic resonance (MR) imaging [13].

This study aimed to investigate associations between demographic, injury related, surgical, preoperative patient‐reported, and subsequent ACL surgery variables for a symptomatic knee and imaging‐based evidence of PTOA 15 years after ACL surgery. It was hypothesised that patient sex, concomitant meniscus injury, initial graft tension, preoperative patient‐reported outcomes (KOOS‐sport, SF‐36 mental health score) and subsequent ipsilateral and contralateral ACL surgery were associated with a symptomatic knee 15 years after surgery. The secondary hypothesis was that these variables were associated with imaging‐based PTOA.

## MATERIALS AND METHODS

This study leveraged the baseline and 15‐year follow‐up data [6] of the surgical patients enroled in a prospective randomised controlled trial (Tension Trial; NCT00434837) evaluating the effects of initial graft tension on outcomes [2, 9, 14, 15]. The Institutional Review Boards of Rhode Island Hospital and Brown University approved the trial, and all subjects granted their informed consent. The Institutional Review Board at University of Connecticut Health Center determined the current analysis to be exempt as it utilised deidentified data from the trial [6, 14].

### Participants and entry criteria

**Figure 1:**
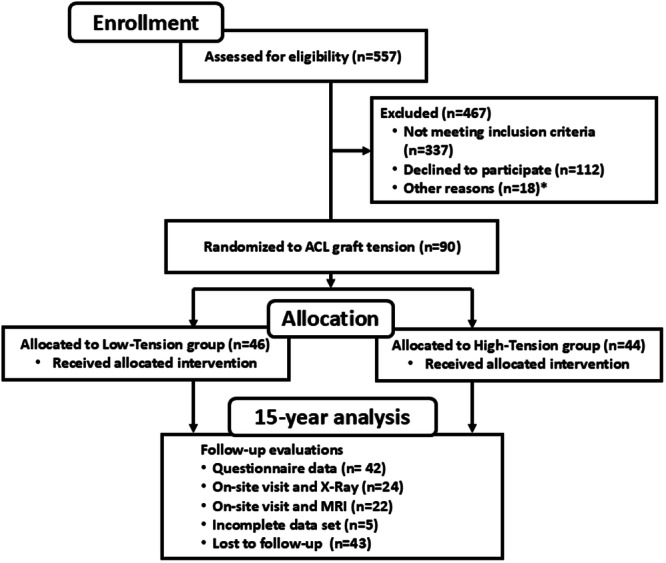
STROBE diagram depicting patient enrolment, lost to follow‐up, and the number of patients undergoing the final analyses at 15 years. *Note that 18 patients were excluded near the time of surgery for other reasons (1 cancelled surgery, 2 opted out of randomisation, 2 had a positive pivot in uninjured knee under anaesthesia, 2 had a partial ACL tear that did not need reconstruction, 1 received an allograft, 1 received a quadriceps tendon graft, 4 presented intra‐operatively with meniscus tears greater than one‐third involvement and 5 presented intra‐operatively with chondral lesions not noted on MRI). ACL, anterior cruciate ligament; MRI, magnetic resonance imaging.

All patients, who presented with a primary isolated unilateral ACL injury in the practices of three surgeons (P.D.F., M.H.J. and R.M.S) were screened using inclusion criteria as previously described [14]. In summary, all male and female patients between 15 and 50 years of age who were candidates for surgical reconstruction with bone‐patellar tendon‐bone or 4‐stranded hamstring tendon autograft (not‐randomised) to treat knee instability were screened. Patients presenting with an ACL injury more than 12 months old, significant concomitant injury to the menisci (defined as a tear involving more than 1/3 of either meniscus) or other ligaments (Grade II or greater) observed on MRI or at the time of surgery, a previous knee injury to either knee, or radiographic or intra‐operative evidence of osteoarthritis, or any metabolic diseases were excluded. Of the 557 patients screened, 355 were excluded as they did not meet the inclusion criteria, and 112 declined to participate (Figure 1). Ninety patients were enroled in the study, with 46 subjects in the low‐tension group and 44 subjects in the high‐tension group [14]. All subjects followed a standardised rehabilitation programme designed to return them to sport by 6 months [5]. At 15‐year follow‐up, 42 patients had complete questionnaire data, and 24 and 22 patients had radiographic and MRI assessments, respectively (Figure 1).

### Surgical technique

Details regarding the surgical technique have been previously reported [6, 14]. The reconstructions were performed using either a bone‐patellar tendon‐bone autograft harvested from the central third of the ipsilateral patellar tendon or a four‐stranded hamstring autograft. The operating procedures were standardised between surgeons, and a transtibial drill guide system was used. For patellar tendon grafts, the bone blocks were secured with metal interference screws [14]. The hamstring tendon grafts were fixed with cortical fixation on the femur and a biodegradable interference screw on the tibia, which was reinforced at the surgeon's discretion with a screw and spiked soft tissue washer [14]. Initial graft tensioning was performed following the ‘laxity‐basedș protocol as described below [4, 14].

### Independent variables

The independent variables included patient sex, the presence of a minor meniscus injury (involving <1/3 of either meniscus), preoperative KOOS‐sport, preoperative SF‐36 mental health score, initial graft tension at time of surgery, and subsequent ipsilateral and contralateral ACL surgery. Two laxity‐based initial graft tension levels were evaluated: (1) setting the initial graft tension to restore normal anteroposterior (AP) laxity at the time of surgery relative to the contralateral uninjured knee (i.e., the ‘low‐tension’ group), or (2) over‐constraining AP laxity by 2 mm relative to the contralateral knee (i.e., the ‘high‐tension’ group) [14]. AP laxity was measured intra‐operatively by an independent examiner using a sterilisable knee arthrometer (KT‐1000S; MEDmetric Corp.).

The KOOS sports and recreation subscore (KOOS‐sport) was collected as a part of the KOOS patient‐reported instrument that evaluates knee symptoms and function [27]. The SF‐36 mental health score was obtained through the SF‐36 instrument that evaluated general health, and the physical and emotional impact of functional limitations [28]. These two patient‐reported variables were selected to serve as independent variables because they were previously shown to be predictive of 7‐year outcomes [29]. All subsequent ipsilateral and contralateral ACL surgeries were recorded over the 15‐year period based on patient reporting with confirmation by the surgical record.

### Dependent variables: Symptomatic knee

The composite KOOS model [13] was used to identify patients with a symptomatic knee at 15‐year follow‐up. Briefly, a KOOS‐QOL value ≤87.5 with two or more of the other subscores meeting the following thresholds (i.e., KOOS‐Pain ≤86.1, KOOS‐Symptoms ≤85.7, KOOS‐ADL ≤86.8 and KOOS‐sport ≤85.0) were indicative of patients with a symptomatic knee [13, 30].

### Dependent variables: Imaging‐based PTOA outcomes

The overall condition of the surgical and contralateral uninjured control joints was assessed using the modified Osteoarthritis Research Society International (OARSI) radiographic grading scale [3, 7, 14, 25]. Using posterior‐anterior and lateral radiographs of both knees, a grade of 0 (normal) to 4 (severe) was assigned to two radiographic features: osteophyte formation and joint space narrowing [3]. In addition, sclerosis, attrition, and ligament calcification were assessed on a dichotomous scale to complete the score. An OARSI score was assigned to both the surgical and contralateral limbs. The difference between the two scores was presented as the OARSI difference score (surgical—contralateral limb) at 15 years. A higher OARSI score difference was indicative of more arthrosis.

Imaging PTOA was also assessed using the Whole Organ Magnetic Imaging Score (WORMS) [14, 24]. The score utilised a set of MR sequences to grade 14 independent features; cartilage signal and morphology, sub‐articular bone marrow abnormality, sub‐articular cysts, sub‐articular bone attrition, and marginal osteophytes evaluated in 15 regions [24]. The condition of the menisci, cruciate and collateral ligaments, synovitis, loose bodies, and peri‐articular cysts were included in the rubric. A WORMS was assigned to both surgical and contralateral limbs and the difference between the two scores (surgical—contralateral) at 15 years was presented as the WORMS difference. An experienced musculoskeletal radiologist (H.C.G) scored all radiographs while blinded to treatment group. A higher WORMS difference was indicative of more arthrosis.

### Statistical methods

Stepwise logistic regression was used to evaluate predictors of the dichotomous measure of a symptomatic knee as defined by the composite KOOS model [13] 15 years post‐surgery. Similarly, stepwise linear regression was used to identify predictors of PTOA based on imaging (WORMS and OARSI difference scores) at 15 years. Stepwise procedures were based on a backwards elimination procedure using *α* = 0.10 for a variable to be retained in the model. This criterion is more conservative with respect to type I error rate than the *α* = 0.15 recommended by Costanza and Afifi for use in stepwise models to identify potential predictors [10]. Variables considered as candidates for inclusion in the models were: patient sex, concurrent meniscus injury at time of injury, preoperative KOOS‐sport, preoperative SF‐36 mental health score, initial graft tension, and subsequent ipsilateral or contralateral ACL surgery. Correlation analyses were also performed to explore the relationships between the two imaging outcome measures and between the imaging outcomes and presence of a symptomatic knee.

The linear regression models for predicting the OARSI and WORM scores were estimated to have 80% power for detecting predictors that accounted for 25% of the variance of the corresponding difference scores (i.e., partial *R* ^2^ = 0.25). The logistic regression model for predicting individuals with a symptomatic knee at 15 years was estimated to have power 80% for detecting explanatory variables with odds ratio of 2.5 per 1 SD increase (or decrease).

All statistical analyses were conducted using commercial software (SAS version 9.4; SAS Institute Inc). Data are presented as means ± standard deviations unless specified otherwise.

## RESULTS

### Patient characteristics (Table 1)

At 15 years post ACL reconstruction, a total of 47 subjects completed follow‐up, of which five were excluded due to missing values for predictors (Figure 1). The mean time between surgery and the follow‐up assessment was 14.8 years (range: 13.3–17.3 years). Of the 42 subjects with complete questionnaires, plain film radiographs and MRIs were obtained from 24 subjects and 22 subjects, respectively. Twenty‐five of the 42 patients presented with minor concomitant meniscal injuries, which were treated with debridement or repair at the time of the index surgery (Table 2). Over the last 15 years, 14.3% of the patients underwent subsequent ipsilateral ACL surgery and 7.1% of the patients had contralateral ACL surgery as previously described [6].

**Table 1:** Baseline characteristics of participants included in the 15‐year follow‐up analysis (*n* = 42).

Baseline characteristics	
Age at time of injury, y	21.2 ± 7.1
Weight, kg	70.5 ± 13.7
Tension group	
Low tension	24 (57.1)
High tension	18 (42.9)
Patient sex	
Female	18 (42.9)
Male	24 (57.1)
Ethnicity	
White, non‐Hispanic	39 (92.9)
Other	3 (7.1)
Graft type	
BPTB	29 (69.1)
4‐ST/G	13 (30.9)
Meniscus tear	
Yes	24 (57.1)
No	18 (42.8)
KOOS‐sport	54.8 ± 20.6
SF‐36 mental health	76.7 ± 12.5

*Note*: All data are expressed as mean ± SD, except for categorical variables which are presented as *n* (%).

Abbreviations: 4‐ST/G, 4‐stranded hamstrings tendon graft; BPTB, bone‐patellar tendon‐bone graft; KOSS, Knee Osteoarthritis Outcome Score; SF‐36, Short Form 36.

**Table 2:** Number of patients with minor meniscal injuries and treatment procedures at time of index surgery for the three primary outcome measures.

Patients with meniscal injuries	Symptomatic knee analysis	OARSI analysis	WORMS analysis
Lateral compartment			
Partial lateral meniscectomy	6	4	7
Lateral meniscal repair	1	1	0
Medial compartment			
Partial medial meniscectomy	5	2	2
Medial meniscal repair	8	2	2
Both compartments			
Partial L/M meniscectomy	1	0	0
L/M meniscal repair	1	1	1
Medial meniscal repair/partial	3	3	3
Lateral meniscectomy			
Total patients	25	13	11

Abbreviations: OARSI, Osteoarthritis Research Society International; WORMS, Whole Organ Magnetic Resonance Imaging Score.

### 15‐year outcome data

Of the 42 patients who were assessed at 15 years, fourteen (33%) presented with a symptomatic knee [6]. For the OARSI radiographic imaging outcome, the average difference in scores between the surgical and contralateral limbs was 1.8 ± 3.9 (*p* = 0.03). The WORMS had an average difference of 11.4 ± 20.1 points between the surgical and contralateral knees (*p* = 0.02). The two measures of imaging PTOA were highly correlated (*r* = 0.79). Interestingly, neither the OARSI radiographic score nor the WORMS data were correlated with a symptomatic knee (*r* = 0.06 and *r* = 0.05, respectively).

### Predictors of a symptomatic knee at 15‐years

**Figure 2:**
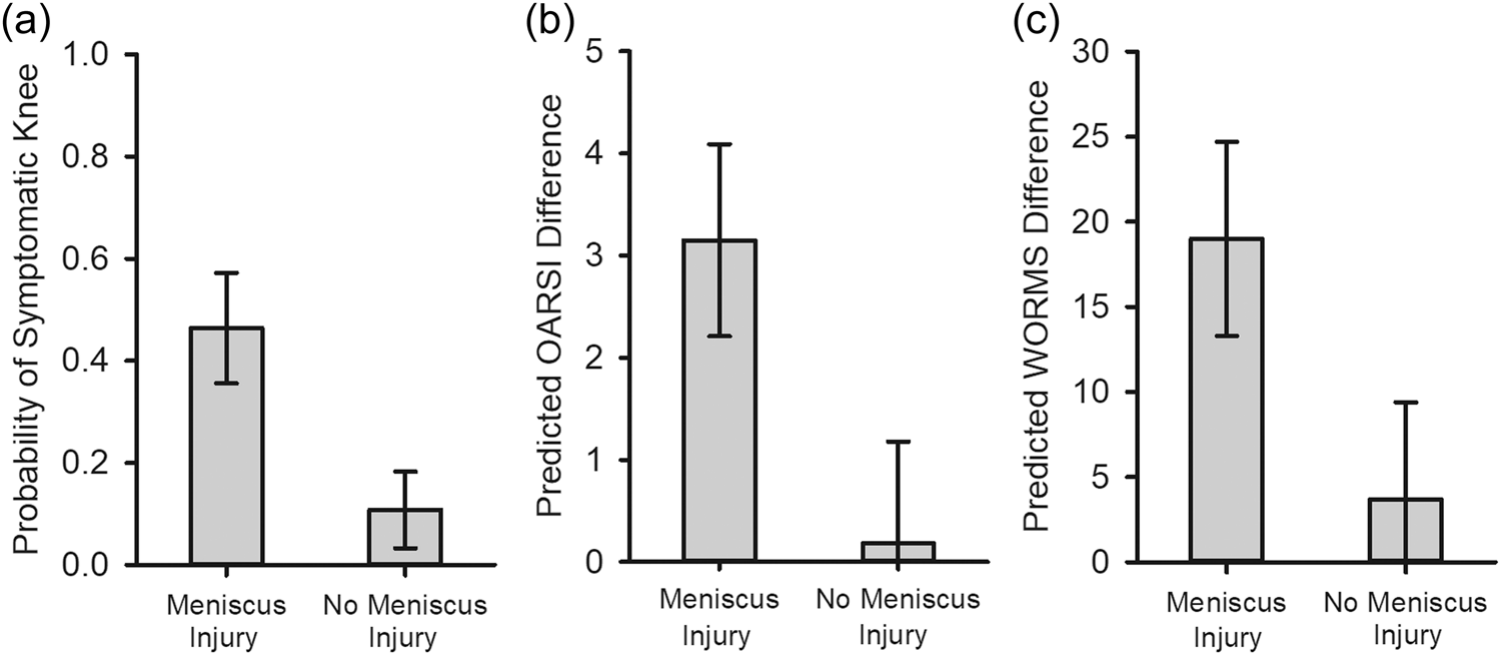
Model predictions related to meniscal injury status at time of the index surgery for presenting with (a) a symptomatic knee, (b) mean difference (surgical – contralateral) in OARSI score, and (c) mean difference (surgical – contralateral) in WORMS at 15‐year follow‐up. Estimates for (a) and (b) are based on patient with average KOOS‐Sports score to isolate the effect of meniscus. The error bars represent the standard error of the mean. KOOS‐sport, Knee Osteoarthritis Outcome Score‐Sports and Recreation; OARSI, Osteoarthritis Research Society International; WORMS, Whole Organ Magnetic Resonance Imaging Score.

There were no significant associations between patient sex, initial graft tension, SF‐36 mental health score, subsequent ipsilateral or contralateral ACL surgery with a symptomatic knee at 15 years after ACL reconstruction. The presence of a minor meniscus tear at baseline increased the odds of a symptomatic knee 7‐fold (Table 3 and Figure 2a). Of the preoperative variables in the model, a higher preoperative (post‐injury) KOOS‐sport was associated with a decreased occurrence of a symptomatic knee 15 years after ACL reconstruction (Table 3). A 10‐point increase in preoperative KOOS‐sport decreased the odds of having a symptomatic knee by 34%.

**Table 3:** Baseline variables significantly associated with a symptomatic knee at 15‐year follow‐up identified via stepwise logistic regression modelling (*n* = 42).

Baseline measure	Coefficient	Standard error	Odds ratio	95% CI	*p* value
Intercept	0.19	1.32			0.88
Meniscus tear	1.96	0.89	7.10	1.24–40.6	0.03
KOOS‐sport^a^	−0.42	0.22	0.66	0.43–1.01	0.06

Abbreviation: CI, confidence interval; KOOS‐sport, Knee Osteoarthritis Outcome Score‐Sports and Recreation.

^a^ Coefficient and odds ratio reflects per 10‐unit increase in the preoperative score.

### Predictors of imaging PTOA at 15‐years

There were no significant associations between patient sex, initial graft tension, SF‐36 mental health score, subsequent ipsilateral or contralateral ACL surgery and imaging PTOA 15 years after ACL reconstruction. The presence of a minor meniscus tear at time of surgery was associated with a greater OARSI difference score 15 years after ACL reconstruction (Table 3 and Figure 2b). Additionally, a significant association between preoperative KOOS‐sport and the PTOA imaging outcome of OARSI difference score was observed (Table 4). A 10‐point increase in the KOOS‐sport was associated with an estimated increase in OARSI difference score between the surgical and contralateral knees by 0.84 points.

**Table 4:** Significant predictors of radiographic OA as determined by the OARSI score difference (surgical – contralateral) 15 years after ACL reconstruction using stepwise linear regression (*n* = 24).

Baseline measure	Coefficient	Standard error	95% CI	*p* value
Intercept	–4.51	2.32		0.06
Meniscus tear	2.97	1.39	0.07–5.87	0.05
KOOS‐sport^a^	0.84	0.36	0.09–1.58	0.03

Abbreviation: KOOS‐sport, Knee Osteoarthritis Outcome Score‐Sports and Recreation.

^a^ Coefficient reflects per 10‐unit change in KOOS‐sport.

No significant associations were found between patient sex, initial graft tension, preoperative KOOS‐sport, SF‐36 mental health score, subsequent ipsilateral or contralateral ACL surgery on imaging PTOA as determined by the WORMS difference. The presence of meniscus tear at baseline was the only significant predictor and was associated with a higher WORMS difference 15 years after ACL reconstruction (Table 5 and Figure 2c).

**Table 5:** Significant predictors of MR imaging OA as determined by the WORMS difference (surgical – contralateral) 15 years after ACL reconstruction using stepwise linear regression (*n* = 22).

Baseline measure	Coefficient	Standard error	95% CI	*p* value
Intercept	3.73	5.72		0.52
Meniscus tear	15.32	8.09	−1.55, 32.18	0.07

Abbreviations: ACL, anterior cruciate ligament; CI, confidence interval; OA, Osteoarthritis; WORMS, Whole Organ Magnetic Resonance Imaging Score.

## DISCUSSION

This study found that the presence of a minor meniscus injury (involving less than 1/3 of either meniscus) at the time of surgery increased the occurrence of both a symptomatic knee and imaging evidence of PTOA at 15‐year follow‐up. While relationships between concomitant meniscus injuries and outcomes following ACL reconstruction have been previously reported [17, 18, 21, 22, 31], the current findings demonstrate that even a minor meniscus injury is predictive of poor outcomes and PTOA. In addition, a higher preoperative KOOS‐sport was associated with a lower occurrence of a symptomatic knee but greater evidence of radiographic PTOA suggesting that a patient's desire to remain active may be a confounding factor. No significant associations were found between patient sex, initial graft tension, SF‐36 mental health score, and subsequent ACL surgery. Understanding factors that are predictive of both a symptomatic knee and imaging evidence of PTOA may identify risk factors that could lead to the design of interventions to prevent a decline in function and quality of life following ACL surgery.

The presence of a minor meniscus injury, even when repaired or debrided, increased the odds of presenting with a symptomatic knee after 15 years by sevenfold. These findings agree with prior reports that meniscectomy is associated with lower KOOS subscores 2 and 10 years after ACL reconstruction [21, 22]. Similarly, a traumatic meniscus tear has also been shown to increase the risk for a symptomatic knee by twofold [13]. These findings were reflected in the current study even though patients with severe meniscus tears (i.e., involving more than one third of the meniscus) were excluded from enrolment [14]. In the current study, a concomitant minor meniscus injury also increased imaging evidence of PTOA, as determined both by higher OARSI and WORMS differences between the surgical and contralateral knees [6]. These results align with a meta‐analysis that also identified a meniscus tear as a risk factor for PTOA 20 years after ACL reconstruction [17]. While some of the meniscus injuries in the current study were treated with debridement or repair, the sample size was too low to evaluate differences in outcomes between untreated, debrided or repaired menisci.

A lower preoperative KOOS‐sport was associated with higher odds for a symptomatic knee at 15 years. Specifically, a 10‐point decrease in preoperative KOOS‐sport increased the odds of having a symptomatic knee by 52% (i.e., 1/OR = 1/0.66 = 1.52). Similar findings were observed at 7‐year follow‐up of the same cohort where a lower preoperative KOOS‐sport increased the odds of having a symptomatic knee [29]. The KOOS‐sport finding suggests that patients with worse function post‐injury and pre‐surgery were more likely to have a symptomatic knee 15 years later. The reasons for this are not completely clear but may be due to injury severity or how patients protected or rehabilitated their knee prior to surgery. This observation is also consistent with the 10‐year outcomes of ACL reconstruction reported by the MOON knee group, wherein a lower post‐injury/pre‐surgery KOOS‐sport was associated with worse KOOS (pain, symptoms, sport, and QOL) subscores at 10‐year follow‐up [22].

The association between preoperative KOOS‐sport and imaging evidence of PTOA needs further consideration. An increase in the preoperative KOOS‐sport was associated with an increase in PTOA as measured radiographically using the OARSI difference score [13]. At 10–12 years after ACL reconstruction, a higher OARSI difference score was also reported for patients from the same cohort with a higher activity level [9]. One could speculate that patients who reported better KOOS‐sport engaged in higher levels of activity, either before and/or after surgery, leading to higher radiographic PTOA at 15 years. Also, patients with a higher KOOS‐sport score may be less symptomatic even though they have more radiographic evidence because they have stronger muscles surrounding the knee, which might minimise symptoms. Future analyses that include an activity matched uninjured control group to account for the confounding effect of activity level may provide important insight about the nuanced relationship between KOOS‐sport and radiographic PTOA.

At 15 years after ACL reconstruction, no significant associations were found between patient sex or initial graft tension with a symptomatic knee or radiographic PTOA. At 10–12 years after ACL reconstruction, patient sex was found to significantly affect the outcome where male patients had significantly worse OARSI scores compared to female patients [9]. The demographic relationship was influenced by initial graft tension as evidenced by a significant interaction between patient sex and initial graft tension at 12 years [9]. Similarly, while a prior study based on the same cohort reported that a lower SF‐36 mental health score increased the odds of having a painful knee 7 years after ACL reconstruction [29], the current study did not find this association at 15 years. In addition, no associations between subsequent ACL surgery with a symptomatic knee or imaging evidence of PTOA were found. It is likely that the loss to follow‐up at 15 years was large enough that the differences observed at 12‐year follow‐up were potentially not detected.

The current study may be the first to describe a relationship between preoperative KOOS‐sport and the occurrence of a symptomatic knee at long‐term (15‐year) follow‐up. A comprehensive model [13] was used to identify patients with a symptomatic knee that was based on the KOOS‐QOL and KOOS‐pain subscores used in prior studies [22, 29, 30] with additional threshold cut offs for other KOOS subscores [13]. The description of the relationship between preoperative KOOS‐sport and imaging PTOA generates further research questions regarding the interaction between preoperative and demographic variables on subsequent PTOA.

In this study, the composite KOOS model was used to determine which patients had a symptomatic knee as a surrogate for symptomatic PTOA. However, no correlation was found between patients with a symptomatic knee and those with imaging evidence of PTOA. It should be noted that the sample size for the composite model outcome was nearly double that of the imaging outcomes which reduced the power of this analysis. Furthermore, the MRI analysis was based on WORMS, one of the earliest semi‐quantitative assessment tools designed to evaluate osteoarthritis progression [24]. Subsequent MRI assessment tools have been developed that could possibly detect more differences [26].

Evaluating the symptoms related to PTOA is challenging since it has been previously shown that patients with imaging evidence of osteoarthritis do not necessarily show symptoms and vice versa [13]. Furthermore, the use of patient reported outcomes to predict symptomatic PTOA has been shown to be variable across instruments [30]. Patient reported outcome measures may capture other knee pathologies that are not directly related to PTOA. Nonetheless, one could postulate that these other pathologies would likely promote knee arthrosis. Therefore, analyses of both the symptomatic and structural domains are important to assess knee arthrosis following surgery.

There are several other limitations to consider. First, patients were enroled between 2004 and 2007 when transtibial femoral tunnels were the norm [32]. However, more anatomic techniques have since been recommended [32, 34], which could possibly improve outcomes. Given that the radiographs were obtained for OA assessment and not tunnel alignment, an accurate tunnel placement assessment was not possible. Furthermore, the standardised ACL rehabilitation regimen prescribed at the time was aimed at returning patients back to sport at 6 months. While functional return‐to‐sport criteria are now commonly used [7], a more conservative rehabilitation strategy would be unlikely to influence outcomes [5, 11, 16]. Second, patients with significant meniscus damage (involving < 1/3 of the meniscus, bucket handle tears) were excluded from enrolment. Therefore, the study may be under‐reporting the strength of the associations between meniscus injury and PTOA. Nonetheless, a 7‐fold increase in having a symptomatic knee at 15 years, even with a minor meniscus injury, was found, which is a clinically interesting finding. The treatment of these minor meniscus injuries included both partial meniscectomy and repair (Table 2). Given the number and location of meniscal procedures, the sample size is too low to compare them directly. Another limitation is that the loss to follow‐up was greater than 50%, which may have precluded us from determining the associations between other preoperative variables that have been reported in prior studies [1, 22, 29, 30]. It should be noted that there were no differences in the demographic variables between the patients with data that were assessed at 15 years with those that were lost to follow‐up or had missing data. As would be expected, the loss to follow‐up increased with time post‐surgery [2, 9, 14]. The small sample size of the current analysis only had sufficient power to detect predictors of PTOA corresponding to relatively large effects sizes. Nonetheless the power was estimated at 80% to detect any explanatory variable explaining approximately 25% of the variability in the imaging outcome variables. Another limitation is that graft type was not randomised, increasing the chance of a selection bias. A recent meta‐analysis showed that bone‐patellar tendon‐bone autografts were associated with a higher incidence of PTOA [33]. While age has been previously shown to increase risk for poor KOOS outcomes [22], the current study did not to include age in the model given the number of variables included in the model and because the age of the cohort at time of surgery was relatively homogeneous (median age of 18 with an interquartile range of 16–24 years). Finally, this study represents a retrospective analysis of data from a prospective randomised controlled trial designed to answer a different research question.

In conclusion, the presence of a minor concomitant meniscus injury, even when treated with partial meniscectomy or repair, was associated with a higher occurrence of a symptomatic knee and radiographic PTOA 15 years after ACL reconstruction. A higher preoperative KOOS‐sport was also associated with decreased occurrence for a symptomatic knee at 15‐year follow‐up. The associations between KOOS‐sport and imaging PTOA were not as clear. A higher preoperative KOOS‐sport was associated with an increased OARSI radiographic difference score but was not associated with an increased MRI based WORMS difference at 15‐year follow‐up.

## AUTHOR CONTRIBUTIONS

Padmini Naga Karamchedu, Braden C. Fleming and Gary J. Badger conceived and designed the study. Padmini Naga Karamchedu, Anika N. Breker, Meggin Q. Costa and Gary J. Badger performed data analyses. Paul D. Fadale, Michael J. Hulstyn, and Robert M. Shalvoy performed the surgeries and participated in data interpretation. Holly C. Gil performed the imaging assessments. Padmini Naga Karamchedu and Braden C. Fleming drafted the manuscript. All authors edited and approved the final manuscript.

## CONFLICT OF INTEREST STATEMENT

Dr. Fleming is a co‐founder of Miach Orthopedics, Inc. and receives royalties. He maintains a conflict‐of‐interest management plan through Rhode Island Hospital. Drs. Fadale and Hulstyn have received travel support from Arthrex. Dr. Shalvoy has received hospitality support from Arthrex, Kairos and Stryker, and consulting fees from Depuy‐Synthes Products. None of these relationships are directly related to this study. All other authors have nothing to disclose.

## ETHICS STATEMENT

Informed consent was obtained following IRB approval (Lifespan IRB 3, Committee number 2013‐05).

## Data Availability

The data are available on ClinicalTrials.gov (NCT00434837) or by direct request to the corresponding author.
